# Comprehensive analysis of the pathogen spectrum and antibiotic resistance profiles in periprosthetic joint infections: a single center retrospective study

**DOI:** 10.3389/fsurg.2025.1566689

**Published:** 2025-03-13

**Authors:** Qianqian Cao, Panlong Fan, Jiawei Feng, Tianmiao Cheng, Xiaoyang Wang, Cheng Cheng, Zhipeng Dai

**Affiliations:** ^1^ Department of Orthopedics, Henan Provincial People’s Hospital, Zhengzhou University People’s Hospital, Zhengzhou, Henan, China; ^2^ Department of Orthopedics, Henan Provincial People’s Hospital, Henan University People’s Hospital, Zhengzhou, Henan, China; ^3^ Center for Clinical Laboratory, The First Affiliated Hospital of Soochow University, Suzhou, Jiangsu, China

**Keywords:** periprosthetic joint infection, pathogen, antibiotic resistance, diabetes, gram-positive cocci, gram-negative bacilli, coagulase-negative staphylococci, staphylococcus aureus

## Abstract

**Objective:**

The objective of this study was to preliminarily examine the demographic profiles, the spectrum of pathogenic bacteria, and the antibiotic resistance patterns among patients with periprosthetic joint infection (PJI), while also offering deeper insights into the microbiological characteristics specifically in diabetic patients with PJI.

**Methods:**

A retrospective analysis of 278 patients diagnosed with PJI from January 2019 to December 2024 at our institution was performed. Demographic characteristics of the patients, the distribution of pathogenic bacteria, and data on antibiotic resistance were statistically analyzed employing the chi-square test and *t*-test.

**Results:**

Gram-positive cocci comprised 56.6% of all pathogenic bacteria, whereas coagulase-negative staphylococci constituted 28.1% of the total. Throughout the study period, a significant decrease was observed in the proportion of rifampicin-resistant coagulase-negative staphylococci (CoNS), from 27.0% to 10.4%. Similarly, a marked decline was noted in the proportion of gentamicin-resistant Staphylococcus aureus, from 50.0% to 15.4%. Conversely, there was a dramatic increase in the proportion of amoxicillin-clavulanate-resistant gram-negative bacilli, from 23.1% to 64.7%. The incidence of fungal infections was notably higher among diabetic patients with PJI compared to their non-diabetic counterparts.

**Conclusion:**

This study demonstrates that the distribution pattern of pathogenic bacteria and their antibiotic resistance profiles among patients with PJI undergoes continuous variation. Moreover, there exist significant differences in the distribution of pathogenic bacteria between those with diabetes and those without diabetes among PJI patients. This serves as a crucial theoretical foundation and empirical support for the rigorous and tailored development of anti-infective treatment strategies for patients with various types of PJI.

## 1 Introduction

Periprosthetic joint infection (PJI) represents one of the most prevalent complications subsequent to hip and knee arthroplasty, serving as a pivotal factor necessitating revision surgery in patients (1–3). PJI is regarded as a catastrophic outcome of arthroplasty, given its consequences of not only prolonging hospital stays and increasing healthcare costs, but also augmenting the risk of long-term disability among patients (4). Notably, diabetes mellitus exacerbates the incidence of PJI by influencing bone metabolism, impeding wound healing, and diminishing immune system function (5–7). The literature has reported an incidence of approximately 2%–2.4% for PJI following total joint replacement in the United States, and 0.33%–1.14% in China (8, 9). Despite the decreasing incidence of PJI with continuous improvements in perioperative management, the overall number of PJI patients continues to escalate due to the rising number of joint replacement surgeries in China, posing a significant challenge to orthopedic surgeons in clinical diagnosis and management (10). Consequently, the prompt and precise identification of causative microorganisms, enabling timely administration of appropriate antibiotic therapy, is crucial in the diagnosis and management of PJI (11–13).

Previous studies have demonstrated significant variations in the distribution of pathogenic microorganisms associated with PJI across different countries and regions, and have highlighted that these microbial profiles, as well as antibiotic resistance patterns, undergo dynamic shifts over time (14–18). Presently, the majority of research efforts are directed towards diagnostic methodologies, risk factors, and prognostic evaluations of PJI (19). Conversely, there is a notable scarcity of studies specifically dedicated to elucidating the microbiological profile of PJI and antibiotic resistance, with the majority of these studies being conducted predominantly in certain countries in Europe and the Americas (20, 21). Considering the direct correlation between alterations in microbial profiles and antibiotic resistance, and the treatment outcomes of PJI, a comprehensive understanding of these dynamic shifts is paramount for the formulation of scientifically rigorous perioperative anti-infective treatment strategies.

The aims of this study were (1) to conduct an in-depth analysis of the microbial profiles and antibiotic resistance characteristics of patients with PJI in China, and (2) to investigate further the microbial distribution characteristics among diabetic patients with PJI. It is anticipated that the findings of this study will serve as a valuable reference for the prevention and empirical treatment of PJI in China.

## 2 Methods

### 2.1 Data source

The study comprised patients with PJI who underwent inpatient treatment at our institution from January 2019 to December 2024. The study adhered strictly to the ethical guidelines outlined in the Declaration of Helsinki (Ethical Principles for Medical Research Involving Human Subjects) and obtained formal approval from the Ethics Committee of Henan Provincial People's Hospital. The study employed retrospective analysis, reviewing solely the medical electronic records of patients without exerting any direct or indirect impact on their clinical treatment. In this study, all participants signed written informed consent forms.

### 2.2 Inclusion criteria

Patients diagnosed with PJI were included according to the criteria established by the Musculoskeletal Infection Society (22). According to these criteria, PJI is considered when one of the following major criteria is present: (1) Two positive periprosthetic cultures with phenotypically identical organisms; (2) The presence of a sinus tract, or when three of the following five secondary criteria are met: (1) Elevated serum CRP (>10 mg/L) or ESR (>30 mm/h); (2) Elevated synovial fluid white blood cell count (>3,000 cells/ml) or a positive leukocyte esterase strip test (++ or +++); (3) Elevated synovial fluid percentage of granulocytes (>80%); (4) A single positive culture; (5) Positive histologic analysis of the periprosthetic tissue, with >5 neutrophils in each of the five high-power fields at 400× magnification.

### 2.3 Data collection and case definition

Over a 6-year period (January 2019 to December 2024), we gathered demographic information, Type 2 diabetes status, other comorbidities, pathogenic bacterial species, as well as antibiotic resistance data for patients with PJI at our institution, utilizing an electronic medical record system. When encountering multiple pathogenic bacteria cultured from the same joint at different time points, priority was given to recording preoperative or intraoperative culture results. Multiple bacterial infections were defined as the concurrent isolation of two or more pathogenic bacteria from periprosthetic tissue and/or synovial fluid. To enhance study accuracy and minimize errors, the 6-year period was divided into two consecutive 3-year intervals (2019–2021 and 2022–2024), during which the microbial profiles and antibiotic resistance characteristics of these two time periods were compared and analyzed. Furthermore, an in-depth exploration of the microbiological distribution characteristics of diabetic patients was conducted by stratifying all PJI patients over the 6-year period into a Type 2 diabetic patient group (DM-group) and a non-diabetic patient group (Control group), based on their diabetes status.

### 2.4 Statistical analysis

The linear-by-linear association chi-square test and the *t*-test were employed to assess the trends in demographic characteristics, comorbidity profiles, the distribution of microorganisms, and patterns of antibiotic resistance among patients with PJI. Statistical significance was established based on a *P*-value threshold of <0.05. SPSS software (version 20.0; IBM Corp., Armonk, NY, USA) was used for performing statistical analyses.

## 3 Results

### 3.1 Analysis of demographic information

From January 2019 to December 2024, a cohort of 278 patients with PJI was enrolled in this study, comprising 133 patients from 2019 to 2021 and 145 patients from 2022 to 2024. Table 1 presents a comprehensive summary of the demographic characteristics of the entire cohort of patients with PJI. The study results indicated no statistically significant variations in gender distribution or infection site among patients with PJI across the two time periods. Notably, patients with PJI during the 2022–2024 period exhibited a significantly greater age compared to those in the 2019–2021 period (*P* = 0.025). Regarding comorbidities, the analysis revealed that the prevalence of hypertension and ischaemic heart disease was markedly elevated in patients with PJI from the earlier time period (2019–2021) compared with those from the later time period (2022–2024) (*P* = 0.007 and *P* = 0.003, respectively). Conversely, no statistically significant differences were noted in the prevalence of diabetes and arrhythmias between PJI patients in the two time periods.

**Table 1:** Patient characteristics of PJI.

Category	2019–2021	%	2022–2024	%	*P*
Number	133		145		
Gender (*n*.%)					
Female	78	58.6	83	57.2	0.813
Male	55	41.4	62	42.8	
Age	64.3 ± 13.3		67.9 ± 9.7		0.025*
Joint (*n*.%)					
Hip	56	42.1	55	37.9	0.479
Knee	77	57.9	90	62.1	
Comorbidities (*n*.%)					
Hypertension	42	31.6	64	44.1	0.007*
Diabetes	37	27.8	47	32.4	0.427
IHD	14	10.5	35	17.9	0.003*
Arrhythmia	37	27.8	50	34.5	0.232

IHD, ischemic heart disease.

* Statistically significant (*P* < 0.05).

### 3.2 Microbiological profiling of patients with PJI

As shown in Table 2, a total of 302 pathogenic microorganisms were isolated and identified from 278 patients diagnosed with PJI in this study, with their distribution detailed therein. The results indicated that gram-positive cocci were the predominant causative organisms among PJI patients, comprising 56.6% of the cases. Among these, coagulase-negative Staphylococcus (CoNS) were the most prevalent, constituting 28.1% of all isolated pathogenic microorganisms. Gram-negative bacilli, fungi, and Mycobacterium species accounted for 14.2%, 9.2%, and 0.3% of all isolated pathogenic microorganisms, respectively. Furthermore, 19.5% of the bacterial cultures yielded negative results. Table 3 presents the status of PJI patients infected by multiple pathogenic microorganisms, with a total of 26 cases. No significant difference was observed in the infection prevalence between the two time periods (10.5% vs. 8.3%; *P* = 0.520). Overall, the distribution of most pathogenic microorganisms remained largely unchanged between the two time periods.

**Table 2:** Characterisation of microbial distribution in patients with PJI.

Germ	2019–2021	2022–2024	Total	*P*
Gram-postive	80 (53.7)	91 (59.5)	171 (56.6)	0.311
CoNS	37 (24.8)	48 (31.4)	85 (28.1)	0.207
Streptococcus aureus	26 (17.4)	26 (17.0)	52 (17.2)	0.917
Streptococci	13 (8.7)	6 (3.9)	19 (6.3)	0.086
Enterococci	2 (1.3)	6 (3.9)	8 (2.6)	0.164
Other	2 (1.3)	5 (3.3)	7 (2.3)	0.267
Gram-negative	26 (17.4)	17 (11.1)	43 (14.2)	0.116
Escherichia coli	12 (8.1)	4 (2.6)	16 (5.3)	0.035*
Pseudomonas aeruginosa	3 (2.0)	4 (2.6)	7 (2.3)	0.729
Klebsiella pneumoniae	4 (2.7)	0 (0)	4 (1.3)	0.042*
Other	7 (4.7)	9 (5.9)	16 (5.3)	0.646
Fungus	18 (12.1)	10 (6.5)	28 (9.3)	0.097
Negative	25 (16.8)	34 (22.2)	59 (19.5)	0.234
Mycobacterium	0 (0)	1 (0.7)	1 (0.3)	0.324
Total	149	153	302	

CoNS, coagulase-negative Staphylococcus.

* Statistically significant (*P* < 0.05).

**Table 3:** Polymicrobial infections.

Germ	2019–2021	2022–2024
GPC/GPC	7	3
GPC/GNB	3	3
GNB/GNB	0	3
GPC/FUN	4	3
Total	14	12

GPC, gram-positive cocci; GNB, gram-negative bacteria; FUN, fungus.

Further analysis revealed a slight increase in the proportion of gram-positive cocci from 2019 to 2021 to 2022 and 2024 (53.7%–59.5%; *P* = 0.311). Among the gram-positive cocci, the proportion of CoNS and enterococci increased slightly in the latter time period (from 24.8% to 31.4%; *P* = 0.207) and (from 1.3% to 3.9%; *P* = 0.164), respectively, whereas the proportion of Staphylococcus aureus (S. aureus) and Streptococcus decreased marginally (from 17.4% to 17.0%; *P* = 0.917) and (from 8.7% to 3.9%; *P* = 0.086), respectively.

In the second time period, the proportion of gram-negative bacilli exhibited a slight decrease (17.4%–11.1%, *P* = 0.116). Among these bacteria, the proportions of Escherichia coli (E. coli) and Klebsiella pneumoniae significantly declined, from 8.1% and 2.7% in the first time period to 2.6% and 0% in the second, respectively (*P* = 0.035, *P* = 0.042). Conversely, the proportion of Pseudomonas aeruginosa remained relatively stable between the two time periods (2.0%–2.6%, *P* = 0.729). In comparison with the first time period, the proportion of fungi decreased slightly in the second (12.1%–6.5%, *P* = 0.097), whereas the proportion of Mycobacterium spp. increased marginally (0%–0.7%, *P* = 0.324). It is noteworthy that the proportion of negative bacterial cultures rose from 16.8% in the first time period to 22.2% in the second (*P* = 0.234), despite this change failing to achieve statistical significance.

### 3.3 Analysis of resistance of pathogenic microorganisms

In this study, the drug resistance of CoNS and S. aureus was analyzed in depth. The results showed that CoNS and S. aureus exhibited the highest resistance to penicillin, with resistance rates of 90.6% and 96.2%, respectively. However, no significant difference was observed in the rate of penicillin resistance between these two bacteria across the two time periods (*P* = 0.7 and *P* = 1, respectively; Table 4). For oxacillin, there was no significant difference in the resistance rate of CoNS between the two time periods (*P* = 0.456), whereas the resistance rate of S. aureus to oxacillin was significantly lower in the second time period compared to the first (53.8% vs. 26.9%, *P* = 0.050). A significant decrease was observed in the rate of CoNS resistance to rifampicin between the two time periods, from 27.0% in the first to 10.4% in the second (*P* = 0.048). Likewise, the resistance rate of S. aureus to gentamicin was significantly reduced from 50.0% in the first time period to 15.4% in the second (*P* = 0.008). Notably, both CoNS and S. aureus demonstrated a decreasing trend in resistance rates to oxacillin, erythromycin, clindamycin, gentamicin, levofloxacin, and rifampicin in the second time period compared to the first. The resistance of Gram-negative bacilli was also analyzed (Table 5). The results indicated that the resistance rate of Gram-negative bacilli to cefoperazone sulbactam decreased from 19.2% in the first time period to 0% in the second (*P* = 0.057). Conversely, the rate of resistance to amoxicillin and clavulanic acid increased significantly, from 23.1% in the first time period to 64.7% in the second (*P* = 0.007). These results have significant implications for directing clinical drug utilization and formulating infection prevention and control strategies.

**Table 4:** Characterisation of drug resistance in gram-positive cocci.

Germ	P	OX	E	CL	GM	LVX	RA	LNZ	VA
CoNS									
2019–2021	33	26	30	24	24	8	10	0	0
(*n* = 37)	89.2	70.3	81.1	64.9	64.9	21.6	27.0	0	0
2022–2024	44	30	37	21	20	15	5	0	0
(*n* = 48)	91.7	62.5	77.1	43.8	41.7	31.3	10.4	0	0
Total	77	56	67	45	44	23	15	0	0
(*n* = 85)	90.6	65.9	78.9	52.9	52.9	27.1	17.6	0	0
*P*	0.7	0.456	0.657	0.055	0.055	0.325	0.048*	-	-
Staphylococcus aureus									
2019–2021	25	14	19	20	13	9	4	0	0
(*n* = 26)	96.2	53.8	73.1	76.9	50.0	34.6	15.4	0	0
2022–2024	25	7	17	17	4	4	3	0	0
(*n* = 26)	96.2	26.9	65.4	65.4	15.4	15.4	11.5	0	0
Total	50	21	36	37	17	13	7	0	0
(*n* = 52)	96.2	40.4	69.2	71.2	32.7	25.0	13.5	0	0
*P*	1	0.050*	0.552	0.363	0.008*	0.113	0.687	-	-

P, penicillin; OX, oxacillin; E, erythromycin; CL, clindamycin; GM, gentamicin; LVX, levofloxacin; RA, rifampicin; LNZ, linezolid; VA, vancomycin.

* Statistically significant (*P* < 0.05).

**Table 5:** Characteristics of drug resistance of gram-negative bacilli.

Germ	GM	CPX	LVX	ATM	C	SCF	AMC	CARBA	TZP
GNB									
2019–2021	5	11	11	6	9	5	6	4	3
(*n* = 26)	19.2	42.3	42.3	23.1	34.6	19.2	23.1	15.4	11.5
2022–2024	5	5	6	6	5	0	11	1	3
(*n* = 17)	29.4	29.4	35.3	35.3	29.4	0	64.7	5.9	17.6
Total	10	16	17	12	14	5	17	5	6
(*n* = 43)	23.3	37.2	39.5	27.9	32.6	11.6	39.5	11.6	14.0
*P*	0.445	0.398	0.649	0.388	0.725	0.057	0.007*	0.348	0.576

GM, gentamicin; CPX, ciprofloxacin; LVX, levofloxacin; ATM, aztreonam; C, cephalosporin III/IV; SCF, cefoperazone and sulbactam; AMC, amoxicillin/clavulanic acid; CARBA, Carbapenems; TZP, piperacillin–tazobactam.

* Statistically significant (*P* < 0.05).

### 3.4 Distribution of pathogenic microorganisms in patients with diabetic PJI

Previous research has demonstrated that the microbial composition of patients with diabetic PJIs undergoes substantial alterations (23). Given this, we conducted an extensive analysis of the microbial distribution patterns in patients with diabetic PJIs (Table 6). The findings of our study indicated that CoNS was the most prevalent pathogen in patients with diabetic PJIs, representing up to 29.5% of cases. The incidence rate of fungal infections demonstrated a notable increase in diabetic PJIs compared to non-diabetic PJIs (*P* = 0.010). It is also worth noting that no cases of Pseudomonas aeruginosa or Klebsiella pneumoniae infections were observed in the cohort of diabetic PJIs investigated in this study.

**Table 6:** Distribution of PJI pathogenic microorganisms in the diabetic and control groups.

Germ	Control group	DM-group	*P*-value	OR	95% CI	
	*n* (%)	*n* (%)				
Gram-postive	120 (51.5)	51 (53.7)	0.720	0.916	0.568	1.478
CoNS	57 (24.5)	28 (29.5)	0.348	0.775	0.455	1.320
Streptococcus aureus	38 (16.3)	14 (14.7)	0.724	1.127	0.580	2.193
Streptococci	16 (6.9)	3 (3.2)	0.193	2.261	0.643	7.948
Enterococci	5 (2.1)	3 (3.2)	0.591	0.673	0.157	2.872
Gram-negative	32 (13.7)	11 (11.6)	0.600	1.216	0.585	2.525
Escherichia coli	14 (6.0)	2 (2.1)	0.137	2.973	0.662	13.34
Pseudomonas aeruginosa	7 (3.0)	0 (0)	0.088	0.970	0.948	0.992
Klebsiella pneumoniae	4 (1.7)	0 (0)	0.200	0.983	0.966	1.000
Fungus	14 (6.0)	14 (14.7)	0.010*	0.370	0.169	0.810
Polybacteria	22 (9.4)	4 (4.2)	0.112	2.372	0.795	7.079

OR, odds ratio; CI, confidence interval.

* Statistically significant (*P* < 0.05).

## 4 Discussion

In this study, we identified a significant trend towards increasing age among patients with PJI, accompanied by a notable rise in the incidence of hypertension and ischaemic cardiomyopathy. This phenomenon appears to be closely associated with the escalating number of elderly patients undergoing arthroplasty in China. Furthermore, upon comparing the two distinct time periods, our findings revealed that the microbial distribution characteristics remained largely unchanged, with the exception of E. coli and Klebsiella pneumoniae. Notably, CoNS emerged as the most prevalent causative species among PJI patients in this study, whereas S. aureus was reported as the most common causative organism in the United States and Taiwan (24, 25). Specifically, PJIs attributed to S. aureus comprised 17.2% of all PJI cases at our institution, which was marginally higher than the 13.0% reported in Europe (24). Conversely, the incidence of PJIs caused by enterococci was relatively low, at 2.6%, concurring with the findings reported by Helou et al. (26). The study conducted by Benjamin et al. highlighted that PJIs caused by enterococci pose greater treatment challenges (27). Given the ongoing evolution of enterococcal drug resistance profiles, there exists an urgent necessity for additional research in this domain. It is noteworthy to mention that previous studies have reported an increasing trend in the incidence of PJIs caused by streptococci. However, at our institution, a decrease in the incidence of PJIs caused by streptococci was observed, albeit this change was not statistically significant (16).

Although gram-positive cocci are the primary causative agents of PJIs, gram-negative bacilli also play a significant role in their pathogenesis. Prior research has indicated that the proportion of PJIs attributed to gram-negative bacilli falls within a range of 5%–20%, which aligns closely with the findings of our study (14.2%) (28, 29). However, the study conducted by Benito et al. revealed an upward trend in the incidence of PJIs caused by E. coli, contrasting with the findings of our study (18). We hypothesize that this discrepancy may arise from the ongoing enhancement of post-operative care following artificial joint replacement at our institution, thereby effectively mitigating the risk of associated infections.

Our research revealed that PJI caused by fungi and mycobacteria comprised 9.3% and 0.3% of cases, respectively. In contrast, prior research indicated that PJI caused by fungi and mycobacteria was less prevalent, with percentages of 5.6% and 2.4%, respectively (30). The incidence of fungal-induced PJI in our study was elevated compared to previous research. We hypothesize that this discrepancy may arise from patients having received broad-spectrum antibiotics at external hospitals prior to admission, altering the flora distribution; alternatively, the relatively small sample size in our study may have influenced the statistical outcomes. Concurrently, the proportion of negative bacterial cultures among PJI patients at our hospital was 19.5%, representing an increase compared to previous national research (15.9%) (10). It is noteworthy that this proportion exhibited a slight upward trend at our hospital. We hypothesize that this phenomenon may be attributed to patients having self-administered broad-spectrum antibiotics before admission, resulting in a corresponding decline in the positive bacterial culture rate. Consequently, further measures are necessary to enhance the positivity rate of bacterial cultures. A robust correlation exists between PJI resulting from multiple microbial co-infections and adverse prognosis (31). In this study, our findings revealed that the number of patients with PJI caused by multiple bacteria in our hospital showed a slight decreasing trend, and the incidence of multiple bacterial infections among diabetic patients diagnosed with PJI exhibited a notably low rate. This observation may be indicative of the heightened awareness and vigilance among arthroplasty patients regarding the prevention and management of PJI, thereby mitigating the risk of multiple bacterial infections.

During the course of our study, a notable decline was observed in the resistance rate of S. aureus to oxacillin, which aligns with the reduction in the prevalence of methicillin-resistant S. aureus among adults in China (32). Compared with previous studies, the methicillin resistance rate of S. aureus in PJI patients in China is significantly lower than the 60% in European countries at present (33). Furthermore, our study revealed a decreasing trend in the resistance rates of CoNS and S. aureus to oxacillin, erythromycin, clindamycin, gentamicin, levofloxacin, and rifampicin. It is suggested that the decline in resistance rates of CoNS and S. aureus to most antibiotics may be attributed to the effective implementation of infection control measures in Chinese clinical settings. In China, at present, we conduct real-time monitoring of nosocomial infections and outbreaks in general hospitals through the Clinical Antibiotic Use and Resistance Surveillance Network (34). This strategy, combined with a computer-assisted electronic prescribing system, has achieved efficient, scientific, and pragmatic management of antibiotic use. Specifically, by optimizing the antibiotic use process, this system has significantly shortened patients' hospital stays, reduced the abuse of medical resources, and effectively delayed the emergence of antibiotic resistance (35, 36). In China, Gram-negative bacilli typically demonstrate high levels of antibiotic resistance (37). Our study found that the susceptibility of Gram-negative bacilli to cefoperazone and sulbactam increased from 80.8% to 100%, whereas their susceptibility to amoxicillin clavulanic acid decreased markedly from 76.9% to 35.3%. Additionally, the susceptibility of Gram-negative bacilli in our study exceeded 70% only for gentamicin, amitranam, cefoperazone/sulbactam, carbapenems, and piperacillin/tazobactam. Notably, the susceptibility of Gram-negative bacilli to ciprofloxacin increased from 57.7% to 70.6%, consistent with the findings reported by Guo et al. (38). When treating PJI, empirical treatment with amoxicillin-clavulanic acid and levofloxacin should be carefully avoided. In contrast, choosing cefoperazone/sulbactam or carbapenems as empirical treatment regimens may achieve better therapeutic effects. However, for the antibiotic treatment of PJI, the most crucial aspect remains the formulation of an individualized and targeted treatment strategy based on the drug sensitivity results of the pathogens and the patient's drug tolerance.

Gram-positive cocci infection constitutes one of the primary causes of both diabetic and non-diabetic PJIs (39). Previous studies have indicated that patients with diabetic PJI are more prone to S. aureus infection compared to those with non-diabetic PJI (40). This susceptibility is typically ascribed to diabetes-induced peripheral neuropathy and vascular damage, which in turn promotes S. aureus colonisation on the skin surface (41). However, the results of this study demonstrated that, although diabetic PJI patients exhibited a slightly higher tendency for S. aureus infection than non-diabetic PJI patients, this difference did not attain statistical significance. We hypothesize that this may be related to preoperative educational interventions for diabetic patients, resulting in a heightened focus on glycaemic control and proactive infection prevention measures during the postoperative period. Our results indicated that when patients with diabetes develop PJI, the proportion of fungal infections is significantly higher than that in non-diabetic PJI patients. Notably, the study by Yuan et al. demonstrated that diabetes compromises the function of macrophages and inhibits their transition from the M1 phenotype to the M2 phenotype, which may be an important reason for the difficult healing of diabetic foot wounds (42). Macrophages eliminate fungal infections through multiple mechanisms, including oxidative killing, phagolysosome acidification, and activation of the Pyrin inflammasome (43, 44). Meanwhile, the hyperglycemic milieu associated with diabetes provides favorable conditions for the proliferation and colonization of fungi (45). Therefore, the increased susceptibility to fungal infections in diabetic PJI patients may be due to the combined effects of a compromised immune response and a hyperglycemic environment. Notably, the cycle of mycological testing is often long. In cases where fungal infection is highly suspected but not yet diagnosed, initial empirical therapy with broad-spectrum antifungal drugs can be considered (46). However, it is surprising that some studies have found that excessive blood glucose can diminish the efficacy of voriconazole and amphotericin B (47). Therefore, for diabetic patients after joint arthroplasty, strict control of blood glucose levels and maintenance of wound hygiene are key measures to prevent the onset of PJI.

Our study has the following limitations: (1) This study is a retrospective study and may have some inherent biases. (2) This study was a single-centre study with a small number of PJI patients enrolled. (3) The cohort of this study consisted of mono-ethnic patients, and caution is needed in generalizing to other ethnicities. Therefore, we need to conduct a multicentre, multiracial, and large-sample study to further confirm our findings.

## 5 Conclusion

During the 6-year study period, we observed no notable alterations in the distribution patterns of pathogenic bacteria, with CoNS consistently serving as the predominant causative agent. Significantly, both CoNS and S. aureus exhibited decreasing levels of resistance to oxacillin, erythromycin, clindamycin, gentamicin, levofloxacin, and rifampicin. Conversely, resistance to amoxicillin-clavulanic acid among Gram-negative bacilli has increased markedly. Similarly, CoNS remained the most prevalent pathogen in studies involving patients with diabetic PJI. Concurrently, we observed a substantial increase in the incidence of fungal infections among diabetic PJI patients. Our findings indicate that the distribution patterns and antibiotic resistance profiles of pathogenic bacteria in PJI patients are in a constant state of flux. Furthermore, there exist differences in the distribution of pathogenic bacteria between patients with diabetic PJI and those without diabetes. By comprehensively considering the drug sensitivity test results of the patient's pathogenic bacteria, their susceptibility to different antibacterial drugs, and their underlying disease conditions, we can customize the most suitable antibiotic treatment regimen for patients with prosthetic joint infection (PJI). This approach not only significantly enhances the treatment effect but also effectively reduces the risk of drug resistance, thereby improving the patient's prognosis.

## Data Availability

The original contributions presented in the study are included in the article/Supplementary Material, further inquiries can be directed to the corresponding authors.
